# Community health workers’ dissemination of COVID-19 information and services in the early pandemic response: a systematic review

**DOI:** 10.1186/s12913-024-11165-y

**Published:** 2024-06-07

**Authors:** Jane Oliver, Angeline Ferdinand, Jessica Kaufman, Nicole Allard, Margie Danchin, Katherine B. Gibney

**Affiliations:** 1 Department of Infectious Diseases, The Peter Doherty Institute for Infection and Immunity, University of Melbourne, 792 Elizabeth St, Melbourne, VIC 3000 Australia; 2 Murdoch Children’s Research Institute, Parkville, VIC Australia; 3 Centre for Health Policy, Melbourne School of Population and Global Health, University of Melbourne, Melbourne, VIC 3000 Australia; 4 Department of Paediatrics, University of Melbourne, Melbourne, VIC 3000 Australia; 5 cohealth, Melbourne, VIC Australia; 6 Department of General Medicine, The Royal Childrens Hospital, Melbourne, VIC 3052 Australia

**Keywords:** Public health, Community health worker, Health promotion, Health services, Community health, Health information

## Abstract

**Background:**

Community health workers (CHWs) had important roles mitigating the impact of the COVID-19 pandemic in vulnerable communities. We described how CHWs supported the dissemination of COVID-19 information and services during the early pandemic response.

**Methods:**

Online article searches were conducted across five scientific databases, with review article reference lists hand searched to identify grey/unpublished literature. Articles were included if they reported on a program that engaged CHWs and aimed to prevent/control COVID-19.

**Results:**

Nineteen relevant programs were identified from 18 included articles. CHWs were widely engaged in the pandemic response, especially in low- and middle-income countries and in vulnerable communities. CHWs’ ability to effectively disseminate COVID-19 information/services was enabled by community trust and understanding community needs. CHWs were often underfunded and required to work in difficult conditions. Pre-existing services incorporating CHWs rapidly adapted to the new challenges brought by the pandemic.

**Conclusions:**

We recommend establishing programs that employ CHWs to disseminate health information and services in communities at-risk of misinformation and poor health outcomes during non-pandemic times. CHWs are well-placed to deliver interventions should an infectious disease outbreak arise. Having pre-existing trusted relationships between CHWs and community members may help protect vulnerable groups, including when outbreaks occur.

**Supplementary Information:**

The online version contains supplementary material available at 10.1186/s12913-024-11165-y.

## Introduction

Community health worker (CHW) is an umbrella term under which many roles fall. CHWs may have many different responsibilities such as providing clinical services, sharing health information, and assisting clients navigating health services [1]. Their prime function is to link their community with the health system [2]. The World Health Organization (WHO) states CHWs should be “…members of the communities where they work, selected by the communities, answerable to the communities for their activities, supported by the health system but not necessarily a part of its organisation, and have shorter training than professional workers.” [1] In this review, we consider CHWs to be health service providers who are first point of contact for consumers at the community level, and are based in communities or at peripheral health posts [3]. When the COVID-19 pandemic began, CHWs rapidly mobilised to mitigate the impact on vulnerable communities [4]. Their COVID-19 duties sometimes came in addition to already substantial workloads [1]. Such duties included supporting COVID-19 cases and close contacts in home isolation, promoting COVID-19 safe behaviours, collecting surveillance data, and distributing facemasks and hand sanitiser. CHWs also promoted, and sometimes provided, COVID-19 testing and vaccination [1, 5]. A recent commentary on CHWs’ roles during pandemics (including COVID-19) noted that CHWs who are equipped, trained, and supported as part of a well-functioning health system can support health service equity and access, and help to keep pandemic impacts in check [1].

Disseminating health information is a core component of many CHWs’ work [6]. CHW programs may be highly effective in facilitating health-related behavioural changes [6, 7]. This is due to CHWs sharing culture, language, and knowledge about the problems their community experiences. Consequently CHWs can approach conversations in culturally appropriate ways, potentially leading to improved adherence to public health advice and clinical treatments [8]. Furthermore, sharing their knowledge and insights about their community with clinical colleagues working in their communities may help improve clinicians’ understanding of their patients [8]. Delivering health education to peers may benefit CHWs themselves by increasing their self-esteem and enhancing their social skills [6]. Despite their ability to support efforts seeking to improve health outcomes, many CHWs are underpaid, inadequately supplied and supervised, and insufficiently supported by their employers and funding bodies. Such inadequacies may reduce their ability to work effectively, including by damaging community members perceptions of CHWs and the value of their work [1].

The impact of programs which involved CHWs in COVID-19 prevention and control appears extensive given CHWs’ potentially important role in reducing transmission. It is unclear what lessons may be learnt from having rapidly established new roles for CHWs in the pandemic response, or how these learnings may help reduce morbidity in future infectious disease outbreaks [1, 9]. We conducted this systematic review to describe how CHWs supported the dissemination of health information and services to communities to prevent or control COVID-19 in the early phase of the pandemic. In particular, we considered the ways in which CHWs supported the COVID-19 response, and identified enablers and barriers to their effectiveness. Where information was available, we reported on CHWs’ impact on community trust in public health strategies and community uptake of COVID-19 testing and vaccination.

## Methods

Key questions considered in this review were:

- To what extent have CHWs been used in the COVID-19 response and in what capacities?
- What are the key facilitators and barriers to CHWs’ effectiveness in the COVID-19 response?
- How have CHWs impacted on community trust in COVID-19 public health strategies?
- How have CHWs impacted on community COVID-19 test uptake?
- How have CHWs impacted on community COVID-19 vaccine uptake?

Articles were included if the program reported was considered to involve CHWs working with community members as part of an intervention aiming to prevent or control COVID-19.

The inclusion criteria was kept fairly broad in order to capture diversity in the ways CHWs contributed to the COVID-19 response. Included articles must:

- Be written in English.
- Have the full text available online.
- Report on a community program aiming to prevent and/or control COVID-19.
- Describe approach/es wherein CHWs supported the dissemination of health information and/or services to communities.
- Describe a program which had been implemented (not a proposal or a protocol).

Online searches for articles (and reports) were conducted on 28 March 2022 using an OVID interface across five scientific databases: Medline, EMBASE, CINAHL, CAB Direct and Web of Science. In addition, Google searches and hand searches of review article reference lists were performed to identify additional eligible reports, including grey or unpublished literature. The search strategy was developed in conjunction with a University of Melbourne Medical Reference Librarian. In brief, keyword searches on each database included: (“community health worker*” or “health concierge” or “peer support”) and (coronavir* or “corona virus*” or “corona pandemic*” or betacoronavir* or COVID19 or COVID* or nCoV or “novel CoV” or CoV 2 or CoV2 or sarscov2 or sars2 or 2019nCoV2 or “wuhan virus”) and (program or programs or programme or programmes). Search terms for each database are detailed in Appendix 1.

Full citations of identified articles were exported to EndNote (version X9) and duplicates were removed. A single reviewer (JO) conducted title and abstract screening. Following this initial screen, articles which had not been excluded underwent a full text review with the reviewer (JO) determining whether the inclusion criteria were met. Reasons for exclusion following a full text review were noted.

Data from included articles were extracted onto a Microsoft Excel data extraction template using an *a priori* framework based around the research questions. Abstracted data included: the study citation, publication date, design, evaluation type, program start date and end date (or if not mentioned, then the date the program was active), setting, type of CHW and COVID-19 training provided, CHW role, core program components, key outcomes, key program facilitators, key barriers, CHWs impact on community trust in COVID-19 public health strategies, CHWs impact on community COVID-19 vaccine uptake, CHWs impact on community COVID-19 test uptake (extracted data are available on request to the corresponding author). Where no information was available for a particular data field, this was noted. Study designs were classified as experimental, cross-sectional, reviews or case studies, and according to methodology (qualitative, quantitative or mixed). Articles that reported on a program without data were classified as “commentaries” and included. When reporting on effectiveness and impact, any evaluation data were considered: outcome data from formal scientific evaluations were prioritised, followed by reported pre- and post-intervention outcome measures (e.g. the proportion of community members vaccinated before and after a CHW information-sharing campaign). Other evidence, including expert opinions and anecdotes were summarised. Findings from included programs were reported as the authors considered relevant to the research questions. Data extraction and analysis ceased when the researchers felt each data extraction field included all relevant information available in the article, and the results table summaries were sufficiently comprehensive.

## Results

**Fig. 1:**
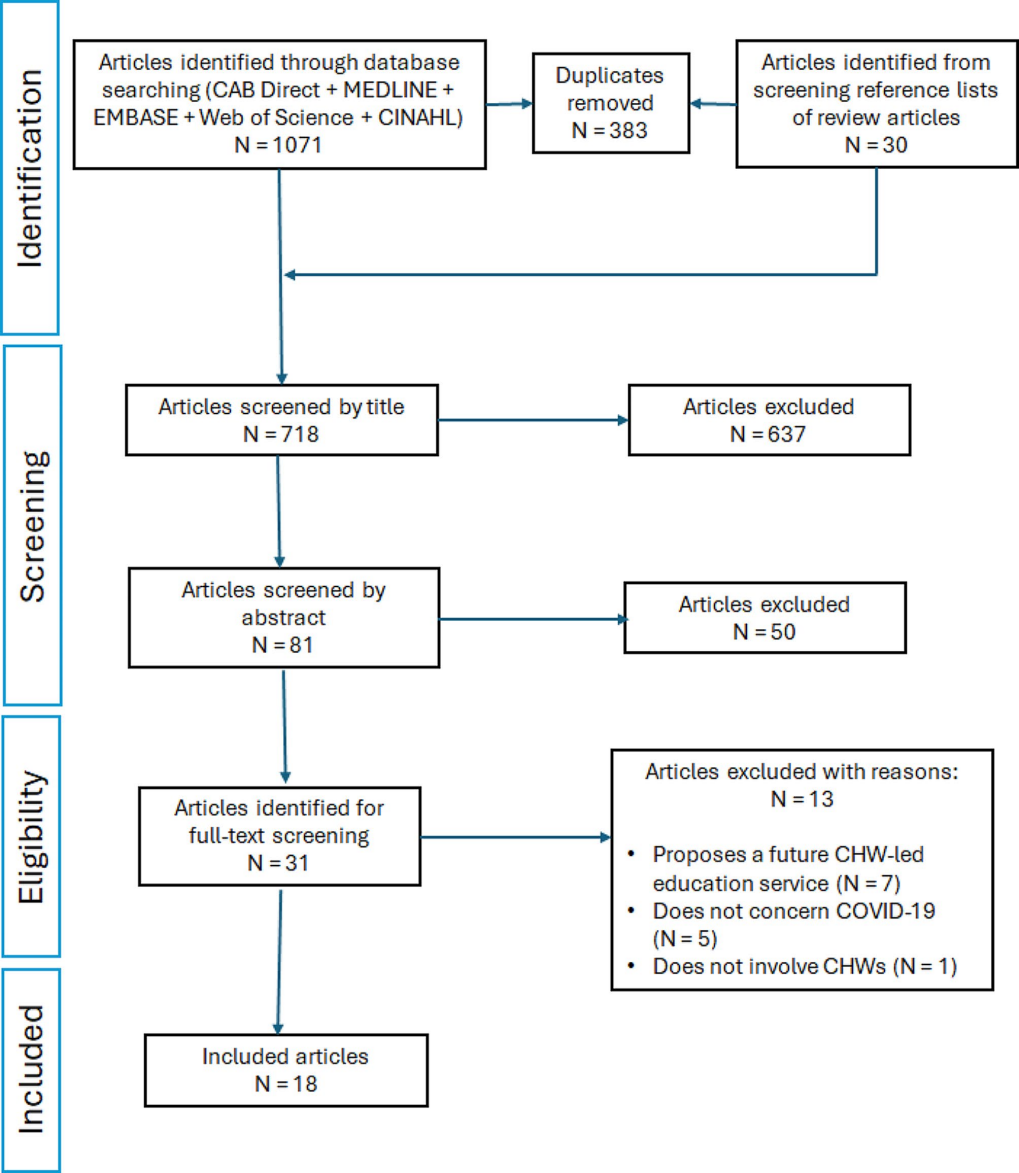
Application of inclusion and exclusion criteria to articles identified in literature searches

Data were abstracted from a total of 18 included articles (Appendix 2). Figure 1 shows how the inclusion and exclusion criteria were applied to articles identified in the literature searches.

Only one article reported an evaluation study. This included pre- and post-intervention data on selected outcomes [10]. All other articles were commentaries [11–25], including one narrative review [26] and two opinion pieces [23, 27]. Ten articles were published in scientific journals [10, 15, 16, 21–24, 26–28], eight were news articles [11–14, 17–20]. The core components and outcomes of these programs (where reported) are outlined in Table 1 and described as follows:

**Table 1:** Description of programs with community health workers supporting COVID-19 prevention and control

Community health worker program	Does program pre-date COVID-19?	Location	Scope	Community health worker intervention	Intervention implementation period	Reach and impact
***Programs with quantitative evaluation***						
**Uganda**						
Village Health Worker (VHW) program	Yes	Kisoro district	52 villages and 48 VHWs	VHWs delivered 4,308 COVID-19 home talks that each lasted 30 min to > 14,000 adults with minimal formal education, and answered their questions. The control group were not visited by VHWs with COVID-19 information[10].	20 April to 16 June 2020	Intervention group scored 30% higher on COVID-19 knowledge test than controls (*p* < 0.0001).
						Significant learning was noted on: COVID-19 symptoms, mechanisms of spread, disease prevention, and risks of mortality, but not about when to go to the hospital with symptoms [10].
						Most participants (82%) in the intervention group reported understanding and valuing information from the home talk more than information they heard via the radio [10].
***Programs without formal evaluation with data***						
**United States**						
Korean Community Services of Metropolitan New York (KSC) program	Yes	New York and New Jersey	Korean American immigrants (numbers not specified)	CHWs partnered with health professionals to share COVID-19 information virtually, answered clients’ questions, promoted testing and vaccination, distributed PPE at outreach sessions [25].	From March 2020	Online meetings and videos reached > 32,000 social media viewers in November 2021.
						> 1,000 people tested for COVID-19 across four events in May 2020.
						Reported increase in COVID-19 vaccine uptake including by offering more than 160 appointments daily, seven days a week [25].
New York –Presbyterian Hospital and the NYU Grossman School of Medicine program	Yes	New York City	50 CHWs worked with underserved culturally and linguistically diverse communities	CHWs contacted socially isolated patients with COVID-19, shared COVID-19 information and connected them with support services [21].	Not stated	From early March to August 2020, CHWs conducted over 9,600 wellness checks via phone, helped nearly 3,400 people enrol in online patient portals and prepare for upcoming telehealth appointments, and conducted virtual health coaching sessions with > 600 patients [21].
South Asian Council for Social Services program	Yes	New York, Queens	> 50,000 clients: underserved South Asians and the broader immigrant community	CHWs disseminated COVID-19 information, distributed masks and sanitiser, promoted testing and vaccination services [25].	Not stated	Between February and May 2021, CHWs provided an average of seven support groups, 187 wellness calls, and 79 counselling sessions/month to community members who had low English-language proficiency.
						During this period 169 vaccinations/month were provided [25].
Unhoused peer ambassador (PA) COVID-19 vaccine outreach program	No	Los Angeles homeless communities	CHWs worked with PAs in pairs (numbers not specified)	PAs introduced CHW to potential homeless clients, distributed food, water, harm reduction supplies and assessed interest in a COVID-19 vaccine, then guided interested people to a nearby mobile vaccine clinic [22].	2020 and early 2021	PAs were valued as vaccine outreach team members and nearly all the 19 CHW participants felt the program was successful [22].
**India**						
Unaccredited community health worker (CHW) training program	Yes	Bihar state	15,000 rural CHWs	A one-day training module taught unaccredited CHWs to: identify possible COVID-19 cases, arrange testing and treatment, monitor cases in home isolation, refer patients with serious symptoms to dedicated health centres, maintain records and co-ordinate activities with the local district control centre [16].	From May 2021	CHW satisfaction: 81/102 (79%) agreed that their training needs were being met and they had received information from reliable sources [16].
						Three-fold increase in people with COVID-19 symptoms referred to primary health centres following the training module (from five people per day to 15 per day, although the time period of observation was not defined) [16].
Accredited Social Health Activist (ASHA) program	Yes	Nationwide	> 900,000 ASHAs nationwide	ASHAs conducted 30–50 home visits/day performing contact tracing, taking travel histories, documenting health profiles, providing home isolation instructions and case monitoring, administering routine medications, maintaining records and sharing COVID-19 information. In various provinces ASHAs also distributed facemasks and performed symptom screening[11, 12, 14, 15].	From March 2020	ASHAs performed COVID-19 symptom screening and checked on high risk people in over 15.9 million households in Karnataka (as of 4 July 2020) [15].
						Over 3,800 people placed in home quarantine with ASHA support in Paravur (as of March 2020) [11].
**Myanmar**						
UN-Habitat Myanmar program	Yes	Yangon (“slum” settlements)	61 CHWs in five settlements	CHWs contacted households, provided COVID-19 information and distributed facemasks [18].	From 2020 (first half)	CHWs contacted > 13,200 households, distributed 102,000 facemasks [18].
**Thailand**						
Village Health Volunteers (VHV) program	Yes	Nationwide	> 1 million VHVs nationwide	VHVs conducted door-to-door visits sharing COVID-19 information, distributed facemasks and hand sanitiser, conducted case follow-up, supported cases and vulnerable people in home isolation, performed temperature screening and contact tracing, organised testing, monitored community gatherings and movements [17, 19, 26].	From January 2020	VHVs visited > 11 million homes in March and April 2020 [17].
						A narrative review concludes VHVs were instrumental in reducing COVID-19 transmission and averting a severe hospital burden [26].
***Programs without data (commentaries)***						
**United States**						
Apicha Community Health Centre program	Yes	New York	Underserved communities, especially Asian immigrants	CHWs shared COVID-19 information, PPE, promoted testing and vaccination [25].	Not stated	Not stated [25]
National Tongan American Society program	Yes	Utah	Pacific Islander communities especially Tongan community	CHWs provided language assistance including for COVID-19 information [25].	Not stated	Not stated(25)
Navajo Nation Community Health Representative (CHR) program	Yes	Navajo Nation	> 100 CHRs serve the Dine’ people	CHRs shared COVID-19 information, performed contract tracing and supported community members, including those in home isolation [23].	Not stated	Not stated [23].
Centre for Pan Asian Community Services program	Yes	Atlanta, Georgia	Underserved communities, especially Asian immigrants	CHWs provided COVID-19 information and testing materials [25].	Not stated	Not stated [25].
The Asian Pacific Community in Action program	Yes	Arizona, Phoenix	Underserved communities, especially Asian immigrants	CHWs addressed COVID-19 vaccine misinformation, facilitated testing and vaccination appointments [25].	Not stated	Not stated [25].
**Bangladesh**						
United Nations High Commissioner for Refugees Rohingya program	Yes	Rohingya refugee settlements	> 1,400 Rohingya refugees trained as CHWs	CHWs went door-to-door sharing COVID-19 information, promoting testing, supporting cases in home isolation.(20)	Late January 2020	Not stated [20].
**India**						
Brihanmumbai Municipal Corporation program	Yes	India, Mumbai	4,000 CHWs	CHWs conducted door-to-door visits to share COVID-19 information and conduct contract tracing [13].	Not stated.	Not reported [13].
**Liberia**						
National Community Health Assistant Program	Yes	Nationwide	CHWs nationwide	CHWs trained to: share COVID-19 information, conduct community surveillance, contact tracing, testing, support cases in home isolation, refer severe cases [27].	Not stated	Not stated [27]
**Zambia**						
Neri/i4life clinic	Yes	Linda township	CHWs serving epilepsy patients	CHWs provided COVID-19 information during home visits, answered questions [24].	From 2020 early March	Not provided [24].

### To what extent have CHWs been used in the response to COVID-19 and in what capacities?

A total of 18 relevant CHW programs were identified from the included articles. Multiple programs were reported from the United States (US; *n* = 9) and India [3], with one program in each of Bangladesh, Liberia, Myanmar, Thailand, Uganda, and Zambia (Table 1).

The only article reporting quantitative data on program effectiveness described a Ugandan program which increased participants’ understanding of COVID-19 safety [10].

Eight programs lacked a formal evaluation, but reports presented data indicating reach and effectiveness. In the US, these programs targeted minority and underserved populations [25, 29, 30]. Two Asian American community program were identified, both used mobile vans staffed with bilingual CHWs to increase access to COVID-19 testing and vaccination [25]. The collaboration between the South Asian Council for Social Services and New York City Health + Hospitals also provided free, walk-in COVID-19 PCR testing and vaccination. CHWs at Korean Community Services provided free COVID-19 testing, vaccination, and antibody screening. Partnerships with Uber and faith-based organisations increased the accessibility and acceptability of these COVID-19 services [25]. Another US program involved multilingual CHW teams delivering health coaching, support, and health system navigation services to underserved culturally diverse communities throughout New York City. This article concluded these CHWs formed a critical bridge between vulnerable communities and the health system [21]. In Los Angeles, a program paired CHWs with experience of homelessness with “peer ambassadors” from homeless communities [22].

A program in India’s third most populous state, Bihar, described a COVID-19 training module delivered to unaccredited CHWs which coincided with a three-fold increase in referrals to primary care [16], although this could reflect an increase in case numbers. India’s nationwide Accredited Social Health Activist (ASHA) Program, which commenced in 2005, was also described [11, 12, 14, 15]. ASHAs are local women trained to work as health educators and promoters in their communities [31, 32]. ASHAs were reported to have a considerable reach and impact on the Indian COVID-19 response [11, 15].

Thailand expanded its nationwide cadre of village health volunteers (VHVs) to over one million personnel. VHVs are a critical point of contact between the community and public health officials [17, 19, 26]. On average, during the pandemic, each VHV was responsible for 10 households [26]. A narrative review suggested that without VHVs, the Thai health system may have been overburdened from COVID-19 [26].

UN-Habitat supported a team of volunteer CHWs in five informal ”slum” settlements in Myanmar’s largest city Yangon. These CHWs’ COVID-19 response was reported to have had considerable reach [18].

The remaining nine programs were described in positive terms by commentaries which included anecdotal evidence and expert opinion [25]. For example, in the Navajo Nation, CHWs are selected by tribal leaders to work in the Navajo-owned-and-run community health program. They receive training from the New Mexico Department of Health [23]. A program in Mumbai, India, involved a workforce of around 4,000 volunteers who were described as the “…backbone of the Brihanmumbai Municipal Corporation’s health workers…” [13].

In Bangladesh > 1,400 Rohingya refugees were trained by the United Nations (UN) to become CHWs. The article identified them as “leading the COVID-19 battle” as a critical community information source [20].

CHWs in Liberia were able to share COVID-19 information through the National Community Health Assistant Program. A March 2020 BMJ Opinion Piece concludes that strategies to rapidly expand healthcare teams with CHWs and develop COVID-19 services were urgently required in Liberia. The authors noted that many countries in sub-Saharan Africa are engaging CHWs in the manner described, and CHWs had been critical in limiting transmission during previous epidemics [27].

In 2019, a CHW program for pediatric epilepsy was launched in a very low-income community, and expanded to include provision of COVID-19 information to clients. The authors stated that although the Zambian Ministry of Health provided guidance on hygiene and social distancing measures, many of the people they served were illiterate and otherwise had limited access to information [24].

### Key facilitators and barriers that affected CHWs’ effectiveness in the COVID-19 response

#### Facilitators

##### Trusted relationships

Communities having trusted relationships with CHWs were consistently described as a major facilitator of the CHWs’ COVID-19 response work. To illustrate, a Thai VHV was quoted as saying, “We know everyone here and we have their trust and confidence.” [17] The selection of CHWs by the community, or by tribal leaders, may have helped to facilitate trust in these relationships [23, 26]. In several cases, articles noted that community leaders (including religious leaders) would endorse CHWs’ health messages and help disseminate their messages [20, 23, 25]. Trust and rapport was often described as being built by the CHWs leveraging their cultural connectedness and shared life experiences. To illustrate, bicultural CHWs were able to connect with community members who were severely affected by the COVID-19 pandemic in New York and engage them with health and support services [21], and unhoused peer ambassadors were valued by vaccine outreach teams working in homeless communities [22]. CHWs in India were sometimes perceived to be doctors by community members, and were the first point of reference for medical ailments [13, 15]. During the COVID-19 pandemic, CHWs were described as, “the most trusted health care provider in the Navajo Nation” [23]. Knowing the community, understanding their needs and having rapport enabled people to “open up” to CHWs [11] and undergo COVID-19 testing without fear of stigma [20]. CHWs’ knowledge of community informed clinicians’ understandings of patients’ needs. Speaking about India’s ASHAs, a spokesperson at Public Services International was quoted as saying, “The skills and the capacity these women have, the way in which they are familiar with each community’s members — the sick, the elderly, the children — the ASHAs are the most likely to know when someone is displaying symptoms of coronavirus, has been traveling abroad or is missing from the home. Without them, doctors will be operating blind.” [14].

#### Dedication to helping community

Articles frequently praised CHWs’ dedication to helping their communities through COVID-19. This dedication often existed in spite of difficult and dangerous working conditions. CHWs conducted home visits despite high community COVID-19 transmission and facing stigma due to being perceived as an infection risk themselves [15, 33]. CHWs quoted in the literature would often speak about the sense of duty and reward they felt helping their communities [12, 20].

#### Responsive service provision

With the exception of the unhoused peer ambassador-CHW program, all programs we identified were already established as health services when the pandemic eventuated (Table 1). Not only did this mean that trusted relationships and reporting mechanisms were already in place, but CHWs already had experience and knowledge of surveillance pathways and community health needs. Pre-existing CHW programs mobilised rapidly in the pandemic response, in some cases following only one or two days of CHW training [10, 13, 23–27]. Having a thorough knowledge of community needs enabled CHWs to provide a responsive service. In the Navajo Nation, CHWs were able to quickly identify the most vulnerable and underserved people to ensure resources made it to families with the highest need [23]. It is possible that having the ability to discuss COVID-19 and ask CHWs questions during education sessions made community members more receptive to their health messages [10, 15, 25].

#### Partnerships with service providers

Community health organisations and CHWs partnered with local public health authorities, clinical service providers and laboratories to co-ordinate the COVID-19 response. CHWs were able to link their community with the partnering organisations/service providers. These partnerships supported public health surveillance and enhanced community access to testing, vaccination and treatment. Partnerships with community organisations enabled other health and wellbeing supports to be delivered [15, 20–23, 25, 26]. To illustrate, in New York, a partnership between the Korean Community Services organisation and Uber enabled less mobile community members to be brought to testing and vaccination providers with encouragement from CHWs, who shared COVID-19 information in language [25].

#### Barriers

##### Program funding and CHW salary

Underfunding was identified as a key barrier to the effectiveness of CHWs’ services in India and Thailand; underfunding may have posed issues in the other included programs but were not reported. ASHAs in India took on COVID-19 work in addition to already heavy workloads. Initially they were not financially compensated for COVID-19 duties. Being considered volunteers, rather than employees, meant ASHAs missed out on employment benefits, including sick leave and carer’s leave. Underfunding may have been related to a lack of recognition from community and government around the value of CHW’s work, which has been noted regarding the ASHA program [11, 14, 15].

##### Occupational risk of infection

ASHAs were at high risk of acquiring COVID-19 through occupational exposure. This risk was compounded by not being provided with adequate PPE and hand sanitiser [11, 14, 15].

##### Occupational risk of stigma

Racism and sexism were sometimes directed against ASHAs; a female and predominantly low-caste workforce. Communities sometimes responded to ASHAs with hostility and in some cases they were subjected to physical violence. This hostility may have been driven by stigma due to ASHAs themselves being perceived as a COVID-19 infection risk. In some cases, ASHAs were stigmatised by their own families due to this perception and excluded from their own homes [11, 14, 15].

##### Communication

In Thailand, VHVs disseminated facemasks to community members which they had needed to hand sew themselves [17]. Reported barriers to effectiveness included a lack of adequate systemic communication between provinces to share and customise good practices for COVID-19 prevention and control. This limited the responsiveness of their service provision. Rural Thai VHVs have stronger community links than their urban counterparts, who may struggle more to connect with communities and subsequently those communities may be less receptive to them [26].

##### CHW burnout

Reports on the ASHA program in India and the Navajo Nation CHW program identified CHWs working long hours, with night and weekend work, as sometimes needed to provide a responsive service. This put CHWs at risk of chronic stress and burn-out [15, 23]. The Navajo report noted that CHWs’ close ties to community meant that they grieved when community members died from COVID-19. Consequently they required time out and support when this happened [23].

### CHWs’ impact on community trust in COVID-19 public health strategies

We noted a lack of empirical data on CHWs’ impact on community trust, with the exception of the Ugandan study which showed that participants valued CHWs’ COVID-19 home talks more than the information they heard on the radio [10]. Anecdotally, trust in the Thai VHVs led to increased trust in local health centers, which in turn empowered people to seek care, even when they were fearful of being stigmatised for having COVID-19 [17, 20]. The Navajo Nation report described a person deciding to disobey public heath orders to collect supplies, but then agreeing to receive deliveries at home after arranging this with a CHW who provided them with much needed reassurance [23].

### CHWs’ impact on uptake of community COVID-19 testing

CHWs’ impact on testing uptake is difficult to ascertain, particularly in times of increasing community cases. Almost all the included CHW programs had a strong focus on CHWs either performing COVID-19 testing themselves [26, 27], or guiding symptomatic people to testing facilities [10–17, 19–21, 23, 24, 26–28]. In Thailand, community compliance with COVID-19 testing and government guidelines was described as, “mostly successful,” which was partly attributed to the VHVs who facilitated testing [26]. The partnerships between the New York Test & Trace Corps and Korean Community Services reportedly resulted in over 1,000 people undergoing COVID-19 testing in May 2020 alone [25]. A WHO staff member is quoted as saying that in Bangladesh refugee settlements (where people had feared being stigmatised if found to have COVID-19), “…the biggest challenge we are facing is convincing people to get tested. The volunteers [CHWs] help us to reach the community and discuss with them the necessity of getting tested and how to prevent further spread of the disease” [20].

### CHWs’ impact on community COVID-19 vaccine uptake

We noted a lack of empirical data demonstrating CHWs’ impact on community COVID-19 vaccine uptake. In Liberia, CHWs were reported to prepare their communities for the introduction of COVID-19 vaccines, although how was not specified [27]. Partnerships between Korean Community Services, the Korean American Physicians Association of New York, Uber, faith-based organisations and vaccine outreach services were reported to have successfully increased COVID-19 vaccine uptake, including among the local senior and immigrant populations. The extent to which uptake was increased is not stated, however the demand was presumably sufficient to warrant vaccine providers making more than 160 appointments available daily with language support, seven days a week [25].

## Discussion and recommendations

CHW programs are now acknowledged as an essential component of a high-performing public health system, regardless of the country’s income level, with CHWs’ critically important roles in the control of outbreaks and noncommunicable diseases recognised [3, 33–35]. The enormous potential of CHWs to extend public health services to vulnerable populations has been well described prior to the COVID-19 pandemic [4, 36]. CHW programs and the roles of CHWs vary greatly across the world, and are influenced by many geographical, historical, cultural, social and health-system factors [4, 37–39]. National governments are increasingly seeking to initiate, scale-up or re-invigorate CHW programs, particularly in low- and middle-income countries. Integrating CHW programs into national health systems can increase the availability of CHW services for vulnerable populations, and enhance CHW supports [4]. To justify investment and maximise community acceptability, ideally regular evaluation would be incorporated into CHW programs, especially when being set up or expanded during a pandemic to account for rapidly changing community needs.

A systematic review which included articles published prior to the COVID-19 pandemic found CHWs’ job satisfaction was a key enabler of their effectiveness [4]. Our review suggests this was also the case during the pandemic, as do two other systematic reviews. CHWs’ job satisfaction is linked with the training they receive, their quality of supervision, and their financial and non-financial work incentives [4, 34, 35]. Drivers of CHW job satisfaction include holding a role which their community respects, recognition of their work, personal growth and career opportunities [4, 34]. Another enabler of CHWs’ effectiveness is their ability to work for a program that is well embedded within their community. ‘Embedded’ here refers to a program which the community accepts and considers locally appropriate [4]. Being adequately supplied for their work is also essential to CHWs’ ability to work effectively. Being inadequately supplied is demotivating for CHWs and can damage the trust communities place in them. Critically, a high workload can impair CHW motivation, satisfaction, efficacy and retention [4, 35]. A second review of (pre-COVID-19) CHW programs in 13 low- and middle-income countries noted CHW role fragmentation and overload was a barrier to their effectiveness. These authors suggested program planners consider re-distributing aspects of CHWs’ roles among workers and volunteers with a range of expertise and training levels [37].

Our review showed that CHWs were widely used in the COVID-19 pandemic response, both in low-middle income countries and in vulnerable communities in high-income countries. Many included programs operated in low-middle income countries where CHWs had pre-existing roles and took on additional COVID-19 related work [11–17, 19, 21, 23, 24, 26]. This likely meant that their other duties were neglected due to a lack of personnel. CHWs’ roles disseminating COVID-19 information were foremost enabled by them being trusted community members who possessed a good knowledge of their communities’ cultures and needs. Despite CHWs being in some cases underfunded and required to work in difficult and dangerous conditions with high workloads and limited PPE, many CHWs showed great dedication to meeting their communities’ needs in the pandemic response. Pre-existing services which incorporated CHWs were able to rapidly adapt to the new challenges wrought by the pandemic and work to mitigate its impact. Critically, communities may have established trust in pre-existing CHW programs which they transferred to CHWs’ COVID-19 work. We observed a lack of empirical evidence of CHWs’ efficacy during the COVID-19 pandemic, with the exception of one study which demonstrated that information from COVID-19 home talks with a CHW was more effectively retained and valued more highly than information heard via the radio [10].

### Limitations

The lack of evaluation studies on CHW program effectiveness during the COVID-19 pandemic likely reflects the time, resources and expertise required to undertake evaluation studies – and the need to focus labour and resources into the pandemic response [40]. Furthermore, given the sudden nature of the pandemic, it is probable that most CHW COVID-19 education programs were not set up to be evaluated.

Despite searching five scientific databases, Google searches and handsearching reference lists of review articles, it is possible that literature was missed. Despite this, we identified similar barriers and supports to CHW’s effectiveness during the pandemic response and two other reviews in the area, notably the need for clear training, adequate resourcing for CHW programs, and de-stigmatisation of CHWs’ work [34, 35]. Importantly literature on indigenous health workers would have been missed by the search strategy used, except if they were specifically referred to as CHWs, as the article discussing the Navajo Nation program did [23]. The contribution that Indigenous health workers have made to COVID-19 control and prevention is considerable. In Australia, early Government engagement with the Aboriginal and Torres Strait Islander Advisory Group on COVID-19 resulted in a number of strategies to prepare these communities for the pandemic. A key preparedness strategy involved health organisations (including Aboriginal-controlled organisations) working with Aboriginal Health Workers to design COVID-19 information and other resources for their local communities [41]. A news article published by the Australian National University reported that the important cultural knowledge and relationships which the Aboriginal Health Workforce had with their communities made them critical to the pandemic response [42]. Several articles discussed Aboriginal Health Workers sharing COVID-19 information with their communities and promoting vaccination [42–44]. This approach to increasing community vaccine uptake is supported by qualitative research, which showed that participants from an Aboriginal community said they would prefer to access COVID-19 vaccination through an Aboriginal Health Worker or a general practitioner who they already knew [45]. As Aboriginal Health Workers do not necessarily take on the same roles as CHWs’ [46], their impacts are considered beyond the scope of this review.

The included studies were not assessed for quality or risk of bias. The majority of the literature detected in our searches pre-dated the widespread availability of COVID-19 vaccines and reflected a health workforce rapidly mobilising to address a new threat. This review is concerned with the early phase of the COVID-19 pandemic and includes articles published as at 28 March 2022. A similar review could be undertaken to elucidate how the role of CHWs changed in the later stages of the pandemic.

The reliance on a single reviewers’ perception is a limitation. A strength of our review is that it considers CHW COVID-19 education programs to identify enablers and barriers in a rapidly changing, novel pandemic, with implications for programs working to address future pandemics.

### Recommendations

In a pandemic response, health programs involving CHW-led information dissemination should foremost acknowledge CHWs’ roles providing reassurance to community members, supporting on-going service access and preventing transmission. In recognising the importance of these roles, employers need to provide CHWs with meaningful renumeration and career opportunities. It is critical that program operators ensure that CHWs are suitably equipped to work effectively at all stages of the pandemic response. By partnering with other service providers, health programs may broaden the range of supports available to communities. Partnering with community leaders may increase uptake of CHWs’ services, especially if leaders are willing to promote their health messages. Through having in-built mechanisms that allow response service provision, health programs can remain relevant in a rapidly changing pandemic response. Such mechanisms include co-designing the program scope with community members and other stakeholders, and recognising that CHWs are themselves stakeholders–therefore program operators need to be receptive to their feedback. Establishing health programs in non-pandemic times allows CHWs to build trust with their communities before a public health emergency response is needed. Detailed recommendations from this review are provided in Table 2.

**Table 2:** Recommendations for implementing programs with community health worker-led information dissemination in an infectious disease outbreak response

Area	Recommendation
**Appreciating community health workers (CHWs)**	• The impacts that CHWs have in infectious disease prevention and control should be recognised by their organisations and program funders.
	• Public health authorities acknowledge that CHW-led peer education may be impactful for preventing infectious disease transmission.
	• Ensuring that CHWs are equipped, trained, supported and remunerated as part of a well-functioning health system enables them to work effectively and mitigates the impacts of an infectious disease outbreak.
	• The impact of the role on CHWs themselves, their professional development, and paths into career opportunities should be considered by program operators.
	• Program operators should incorporate CHW feedback into program evaluations.
**Partnerships**	• By working closely with local public health authorities, CHWs and their organisations can ensure that communities’ needs are understood by policymakers and are met in a timely manner.
	• With their deep understanding of the community, CHWs are well-placed to advise stakeholders on community matters.
	• Clear management and reporting pathways, with clarity around the scope of the CHW role, is likely to improve the work environment.
	• Building mutually supportive relationships between CHWs and community leaders will support service uptake.
	• Bodies that govern and fund CHW programs ought to make an ongoing commitment to providing local people with employment opportunities, including people without formal qualifications.
**Responsive service provision**	• CHW programs should be established in non-pandemic times, to allow rapid and responsive service provision as outbreaks develop.
	• Outbreak responses would be supported by existing CHW programs incorporating an ability to rapidly train and deploy additional CHWs while maintaining usual services.
	• As part of outbreak planning, the scope of the CHW role needs to be co-designed by community so it addresses their priorities and needs.
	• Employing CHWs with strong links to community enhances health service reach through shared connections. This in turn supports the collection of complete and accurate surveillance data and may help mitigate occupational stigma.
	• CHW programs should include planned periodic evaluation co-designed by community with clear outcome measures and a mechanism to gain community feedback.

## Conclusion

Residents of vulnerable communities are at-risk of misinformation and poor health outcomes. CHWs can help protect these communities. Establishing and embedding co-designed programs that employ CHWs during non-pandemic times creates conditions which permit rapid and responsive service provision during outbreaks. Well-embedded CHW programs may help optimise community health, and are important to consider when conducting pandemic preparedness planning.

### Electronic supplementary material

Below is the link to the electronic supplementary material.

Supplementary Material 1

Supplementary Material 2

Supplementary Material 3

## Data Availability

Availability of data and materials: Data from included articles analysed in this review are available in Appendix 2. All other data generated by this review are available from the corresponding author on reasonable request.

## Abbreviations

ASHA: Accredited social health activist
CALD: Culturally and linguistically diverse
CHW: Community health worker
COVID: 19-Coronavirus disease of 2019
PPE: Personal protective equipment
US: United States of America
WHO: World Health Organization
VHV: Village health volunteers
