# Supplementary material for: Community health workers’ dissemination of COVID-19 information and services in the early pandemic response: a systematic review
Source: BMC Health Serv Res. 2024 Jun 7;24:711. doi: 10.1186/s12913-024-11165-y (PMC11161953; doi:10.1186/s12913-024-11165-y)

**Ovid Medline and EMBASE Search Strategies**


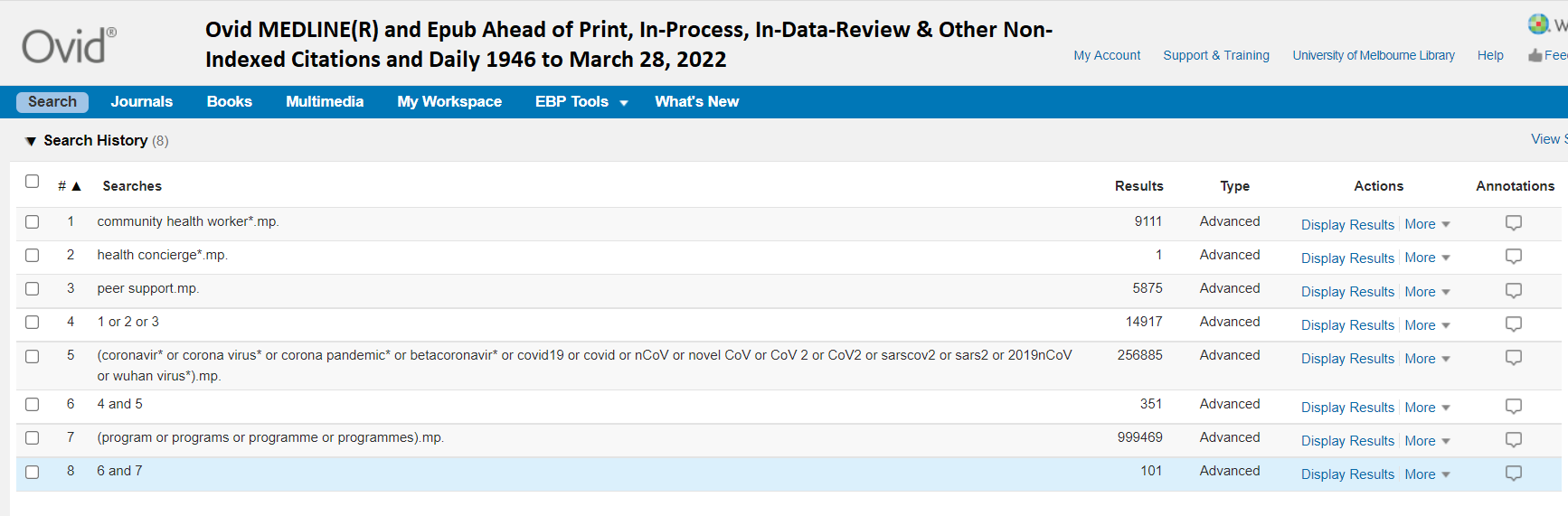


**CINAHL Search Strategy**


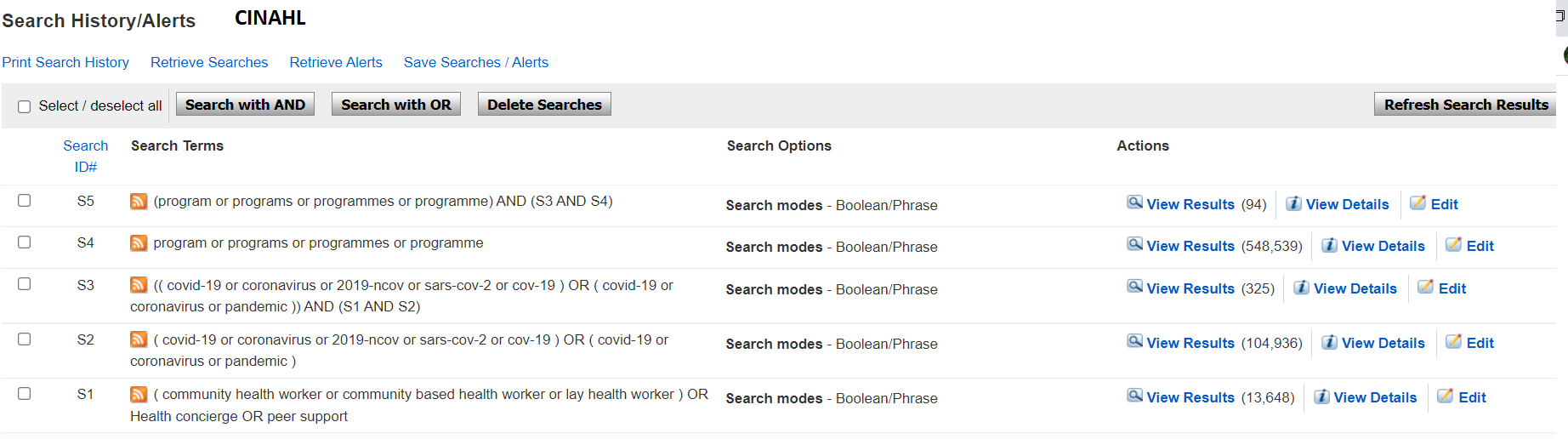


**CAB Direct Search Strategy**


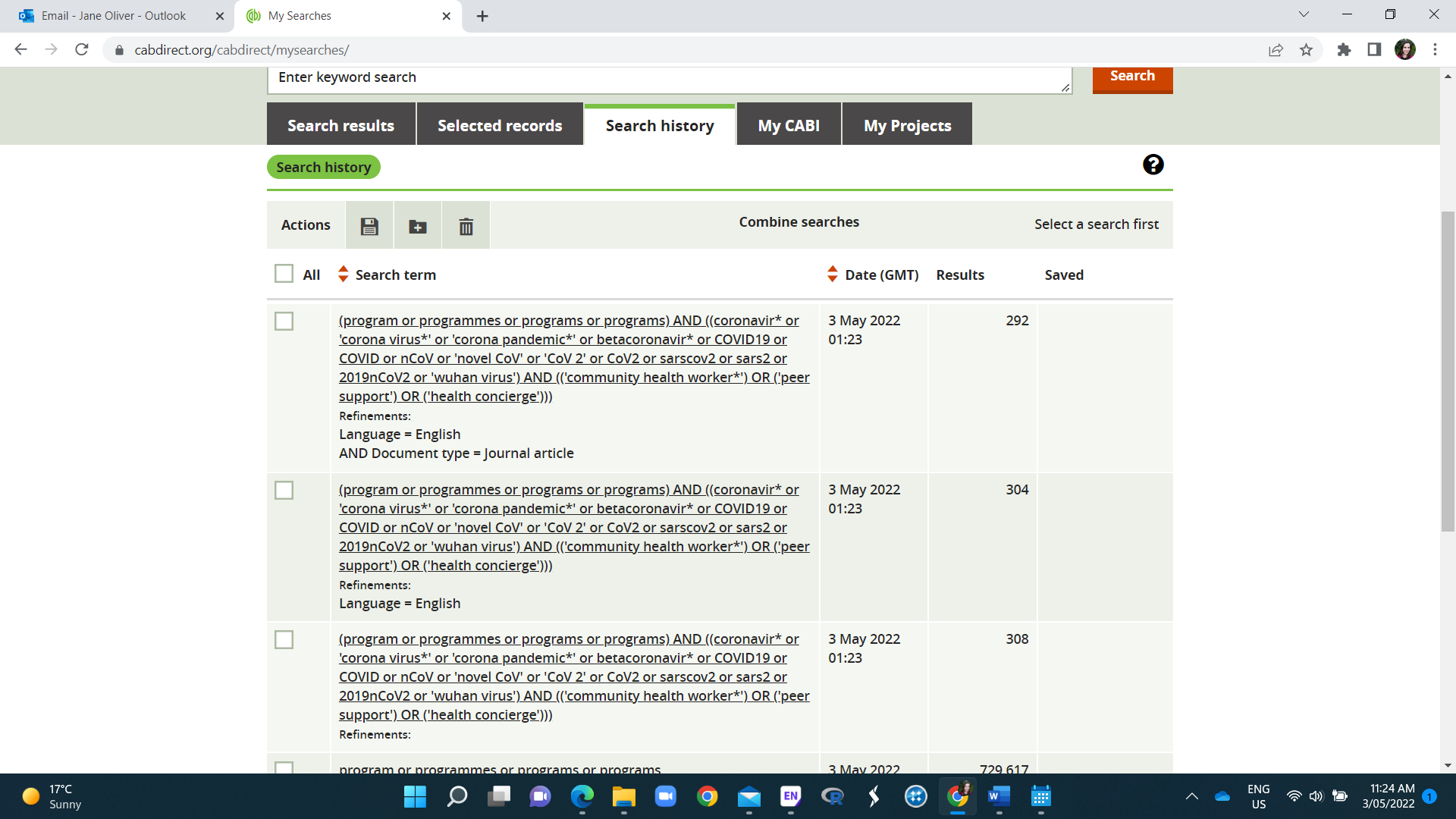


**Web of Science Search Strategy**


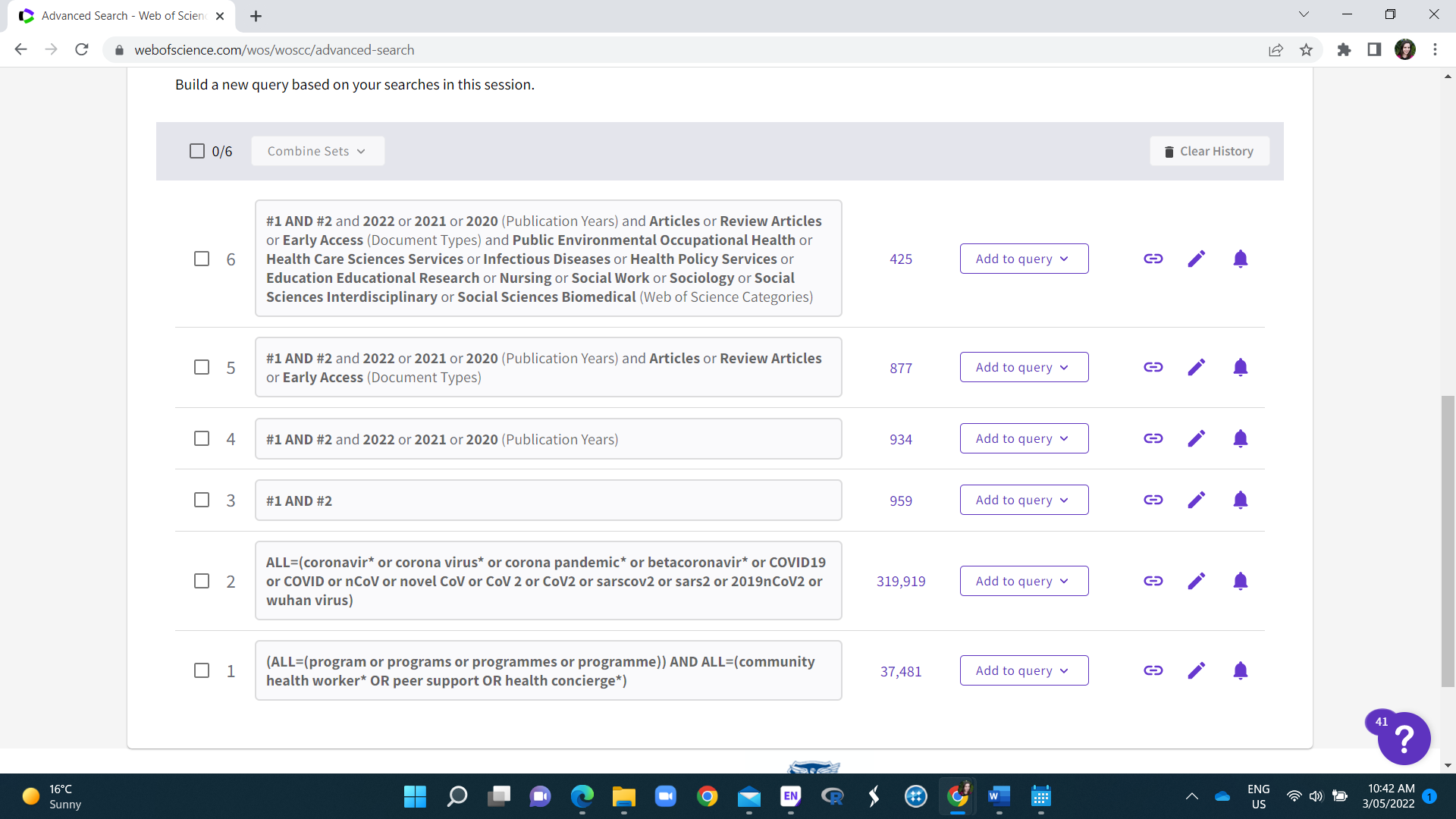

Supplement: Supplementary file 1 — Supplementary Material 1 [file 12913_2024_11165_MOESM1_ESM.docx]
